# The proteasomal and apoptotic phenotype determine bortezomib sensitivity of non-small cell lung cancer cells

**DOI:** 10.1186/1476-4598-6-73

**Published:** 2007-11-17

**Authors:** Jens Voortman, Agnieszka Chęcińska, Giuseppe Giaccone

**Affiliations:** 1 Department of Medical Oncology, VU University Medical Center, 1081HV Amsterdam, the Netherlands; 2 Department of Dermatology, University of Michigan, 4217 Comprehensive Cancer Center, 1500 East Medical Center Drive Ann Arbor, MI 48109-0314, USA; 3 Medical Oncology Branch, National Cancer Institute, National Institutes of Health, 10 Center Drive, Bethesda, MD 20892-1906, USA

## Abstract

Bortezomib is a novel anti-cancer agent which has shown promising activity in non-small lung cancer (NSCLC) patients. However, only a subset of patients respond to this treatment. We show that NSCLC cell lines are differentially sensitive to bortezomib, IC_50 _values ranging from 5 to 83 nM. The apoptosis-inducing potential of bortezomib in NSCLC cells was found to be dependent not only on the apoptotic phenotype but also on the proteasomal phenotype of individual cell lines. Upon effective proteasome inhibition, H460 cells were more susceptible to apoptosis induction by bortezomib than SW1573 cells, indicating a different apoptotic phenotype. However, exposure to a low dose of bortezomib did only result in SW1573 cells, and not in H460 cells, in inhibition of proteasome activity and subsequent apoptosis. This suggests a different proteasomal phenotype as well. Additionally, overexpression of anti-apoptotic protein Bcl-2 in H460 cells did not affect the proteasomal phenotype of H460 cells but did result in decreased bortezomib-induced apoptosis. In conclusion, successful proteasome-inhibitor based treatment strategies in NSCLC face the challenge of having to overcome apoptosis resistance as well as proteasomal resistance of individual lung cancer cells. Further studies in NSCLC are warranted to elucidate underlying mechanisms.

## Findings

Intracellular protein metabolism involves both synthesis as well as degradation of proteins. The vast majority of proteins is selectively degraded by the ubiquitin-proteasome system (UPS). The 26S proteasome complex is composed of a 20S "core", a large protein complex that harbours the proteolytically active sites, and 19S "caps", which play a role in the recognition of poly-ubiquitinated protein substrates, targeted for degradation. Poly-ubiquitination involves covalent ligation of the target protein, by the sequential action of three enzymes, to a chain of ubiquitin molecules [1].

Growing awareness of the pivotal role of the UPS in normal cell physiology as well as in (malignant) disease propelled the development of proteasome inhibitors for therapeutic applications [2-4]. Bortezomib (Velcade) is the first clinically approved small molecule proteasome inhibitor. It reversibly binds and inhibits the chymotryptic-like proteolytic activity of the proteasome, localized within the β_5 _subunit of the 20S core. This results in disturbance of intracellular protein homeostasis by accumulation of poly-ubiquitinated proteins. Among other effects, this can trigger apoptosis, with a relative selectivity for malignant as opposed to normal cells [3].

Lung cancer is the most common cause of cancer-related death in the world [5]. Non-small cell lung cancer (NSCLC) consists of epithelial tumors, accounting for approximately 80% of lung carcinomas. Clinical studies showed promising activity of bortezomib in a subset of patients with non-small cell lung cancer (NSCLC) [6,7]. So far the exact molecular mechanism of resistance of NSCLC to bortezomib remains unclear [8-11]. Recently, adaptation of leukaemia and lymphoma cells to continuous exposure of bortezomib was reported to result from increased expression and altered subunit composition of the proteasome [12,13]. Furthermore, resistance was shown to be associated with increased expression of anti-apoptotic Bcl-2 family member proteins and heat shock proteins [14-17]. In this study, we examined the effects of bortezomib in a panel of NSCLC cell lines. We observed differential activity of bortezomib in NSCLC cells regarding growth inhibition, the proteasome activity profile and apoptosis induction.

**Figure 1:**
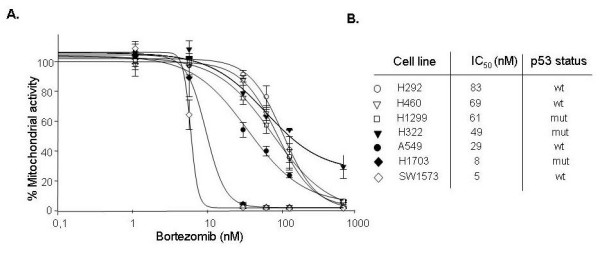
**Growth inhibition by bortezomib in NSCLC cells**. (A) Growth curves (MTT assays) of NSCLC cells treated with different concentrations of bortezomib. Mean of at least three independent experiments, SD. (B) IC_50 _concentrations and p53 status of individual cell lines. Wt: wild type; mut: mutated.

We first determined the anti-proliferative effect of a concentration range of bortezomib by MTT assay in a panel of NSCLC cell lines (Figure 1A). We observed differential sensitivity to bortezomib-induced growth inhibition, with IC_50 _values ranging from 5 to 83 nM. The p53 status did not correlate with sensitivity (Figure 1B). Next, to establish the apoptosis inducing potential of bortezomib, a sensitive cell line, SW1573, and a resistant cell line, H460, were selected for treatment and subsequent PI staining-based FACS analysis to determine the subG_1 _apoptotic fraction of the cell population. Additionally, the fractions of cells in the G_1_, S or G_2_M phase of the cell cycle were assessed.

**Figure 2:**
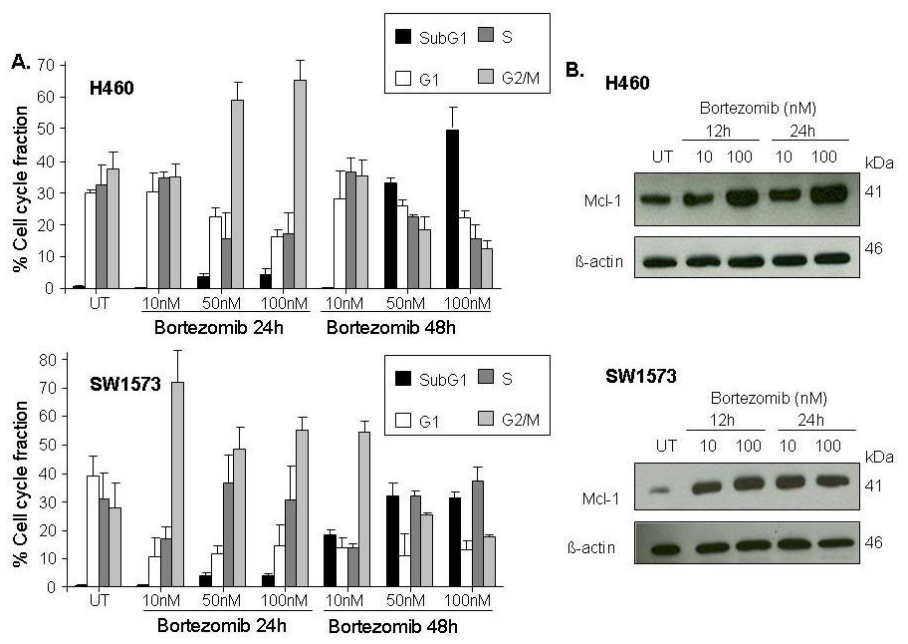
**Induction of apoptosis and cell cycle arrest by bortezomib**. (A) Effect of different concentrations of bortezomib on cell cycle progression of H460 and SW1573 cells upon 24 or 48 hours of exposure. Mean of at least three independent experiments, SD. Ut: untreated. (B) Time and concentration course Western blot analysis of total cell extracts of H460 and SW1573 cells showing the effect of treatment with bortezomib on the expression level of Mcl-1.

As shown in Figure 2, upon exposure of SW1573 cells to a low concentration of bortezomib, 10 nM, cells went into G_2_M cell cylce arrest, whereas H460 cells remained predominantly in the G_1 _phase of the cell cycle. This finding suggested that the proteasome activity in H460 cells is not inhibited at 10 nM of bortezomib, as proteasome inhibition typically results in G_2_M arrest [18,19]. Furthermore, G_2_M arrest in SW1573 and H460 cells was associated with increased expression of Mcl-1, which is known to be upregulated upon proteasome inhibition [15,20]. As shown in Figure 2, Mcl-1 is only upregulated in H460 cells when exposed to bortezomib at a high concentration (100 nM), whereas Mcl-1 is upregulated in SW1573 cells also at a low concentration of bortezomib (10 nM). Concordantly, treatment with bortezomib 10 nM for 48 h led to significant apoptosis in SW1573 cells (18%), but not in H460 cells. However, at a higher dose of bortezomib (50 to 100 nM), both SW1573 and H460 cells went into G_2_M arrest, resulting after 48 h in more apoptotic H460 cells (49%) compared to SW1573 cells (32%). Thus, upon effective induction of G_2_M cell cycle arrest, the apoptosis inducing potential of bortezomib was more pronounced in H460 cells compared to SW1573 cells, indicating a different apoptotic phenotype. In contrast, SW1573 cells went into G_2_M arrest at a lower threshold concentration of bortezomib than H460 cells, also indicating a different proteasomal phenotype of these two cell lines.

In order to further evaluate the mechanism of resistance to bortezomib, proteasome activity was measured upon treatment with various concentrations of bortezomib in H460, SW1573 and H460-Bcl-2 cells. H460-Bcl-2 cells, overexpressing anti-apoptotic factor Bcl-2, have a more apoptosis resistant phenotype compared to the parental H460 cells [15].

**Figure 3:**
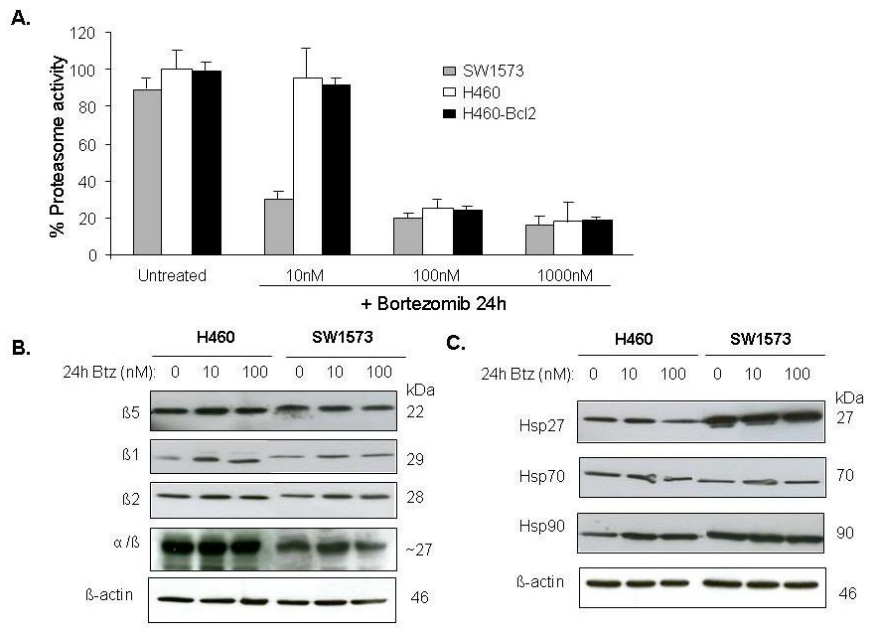
**Proteasome activity and expression of catalytic subunits upon bortezomib treatment**. (A) Intracellular proteasome activity in H460, H460-Bcl-2 and SW1573 cells treated with a concentration range of bortezomib. Mean of at least three independent experiments, SD. (B) Time and concentration course Western blot analysis of total cell extracts of H460 and SW1573 cells showing the effect with treatment with bortezomib on the expression level of the 20S proteasome core (α/β), individual catalytic subunits: β_1,β2_, β_5 _and (C) heat shock proteins Hsp27, Hsp70 and Hsp90.

As shown in Figure 3, the basal level of proteasome activity was about 10% lower in SW1573 cells compared to H460 and H460-Bcl-2 cells. Furthermore, at 10 nM of bortezomib, proteasome inhibition occurred exclusively in SW1573 cells. In contrast, at 100 nM of bortezomib the level of proteasome inhibition was similar in all cell lines. Overexpression of Bcl-2 did not alter the basal proteasome activity nor its inhibition induced by bortezomib [15].

These observations confirmed a differential proteasomal phenotype comparing H460 and SW1573 cells and indicate that the proteasomal phenotype is independent of the apoptotic phenotype.

Bortezomib predominantly inhibits the chymotryptic-like activity of the 20S proteasome, harboured in the β_5 _subunit, and to a lesser extent the caspase-like activity harboured in the β_1 _subunit [21,22]. We therefore next evaluated whether the relative sensitivity for proteasome inhibition of SW1573 cells correlated with a decreased expression level of the 20S proteasome and its individual catalytically active subunits, β_1_, β_2 _and β_5_. As shown in Figure 3B, the expression level of the β_1_, β_2 _and β_5_subunits was slightly increased in H460 cells compared to SW1573 after 24 h exposure to bortezomib (10, 100 nM). Additionally, exposure to bortezomib induced expression of subunit β_1 _in H460 cells as compared to untreated control. In agreement with our results, increased expression of proteasome subunits, as well increased (basal) proteasome activity, was recently related to resistance to proteasome inhibitor treatment [12].

To examine the possibility that apoptotic factors might explain the differential sensitivity for apoptosis induction once the proteasome has been effectively inhibited, we assessed the expression of heat shock proteins, such as Hsp27, Hsp70 and Hsp90 upon exposure of H460 and SW1573 cells to bortezomib. Notably the expression of Hsp27, but also Hsp90, was higher in SW1573 cells than in H460 cells, correlating with less pronounced apoptosis in SW1573 cells. The expression levels of Hsp70 was similar in both cell lines.

NSCLC cell lines display differential sensitivity towards the proteasome inhibitor bortezomib. For effective induction and execution of apoptosis at least two conditions must be met. First, the dose of bortezomib must be above the threshold necessary to inhibit the proteasome activity in individual NSCLC cells. We showed that this threshold concentration differs among non-small cell lung cancer cells. Typically in clinical studies, proteasome inhibition in peripheral blood mononuclear cells is taken as a surrogate pharmacodynamic marker. These results indicate however that this might not be a fair estimate of what is occurring in the actual tumour tissue, as the proteasomal phenotype of individual tumours is subject to significant variability [23,24].

Secondly, upon inhibition of the proteasome, an intrinsic or acquired resistance to apoptosis must be overcome, the apoptotic phenotype being variable in different NSCLC cell lines. In this regard, apoptosis resistance does not directly correlate with resistance resulting from a differential proteasomal phenotype, as was shown in SW1573 cells, which were more sensitive to proteasome inhibition, but more resistant to apoptosis than H460 cells. Additionally, H460 cells overexpressing anti-apoptotic Bcl-2 showed a similar proteasomal phenotype as did wild type H460 cells. Increased apoptosis resistance of SW1573 cells compared to H460 cells might result from increased expression of Hsp27 in SW1573 cells, which has been postulated to be a resistance factor for bortezomib-induced cell death [14].

It was recently suggested in a mice model of colon carcinoma, that potentiation of the anticancer activity of bortezomib by combination with TNF-α resulted from reduced expression of proteasome subunits and inhibition of Hsp27 [25]. Breast carcinoma cells were shown to be more resistant to treatment with bortezomib than normal breast cells, despite a higher level of feedback proteasome upregulation in the normal cells. The authors suggested resistance of the cancer cells compared to the normal cells was primarily due to a defective pathway of bortezomib-induced apoptosis in the cancer cells [11]. Another report suggested resistance to bortezomib can occur in lymphoma cells through an altered proteasomal phenotype coinciding with increased expression and altered subunit composition of proteasome [12]. Our preliminary results indicate that also H460 cells can adapt to continuous incremental exposure of bortezomib (data not shown).

Additionally, we recommend an assay allowing cell fraction measurement, such as PI-staining based FACS analysis, over an anti-proliferative assay, such as MTT, to characterise the cytotoxic effects of proteasome inhibitors. Cell fraction measurement by PI-staining based FACS analysis is informative about the apoptosis inducing potential (subG_1 _fraction) and inhibition of proteasome activity (G_2_M arrest). In contrast, a low IC_50_(e.g. SW1573 cells), as determined by MTT assay, does not necessarily correspond to enhanced apoptosis induction in a certain cell line, compared to a cell line with a higher IC_50 _(e.g., H460 cells).

Our preliminary results show that the proteasomal as well as apoptotic phenotype determines bortezomib sensitivity in NSCLC cells. There is a preclinical rationale to combine proteasome inhibition with pro-apoptotic agents as well as agents promoting a more favourable proteasomal phenotype to overcome this resistance [20,26].

## Materials and methods

### Cell lines and drugs

NSCLC cell lines H292, H460, H1299, H322, A549, H1703 and SW1573 were obtained from the American Type Culture Collection (Manassas, VA, USA) and cultured in RPMI1640 or, SW1573 only, DMEM (Cambrex Bioscience, Verviers, Belgium). H460 cells stably overexpressing Bcl-2 (H460-Bcl-2) were generated previously [27]. Culture mediums were supplemented with 10% FCS, 100-units/ml penicillin, and 100-μg/ml streptomycin (Invitrogen, Breda, The Netherlands). Bortezomib (Velcade™) (Millennium Pharmaceuticals Inc. Cambridge, MA, USA) was dissolved in DMSO.

### Growth inhibition assay

Growth inhibition was determined 72 h after treatment with a concentration range of bortezomib, by MTT assay, as described previously [15]. Results are presented as percentage of survival taking the control (untreated cells) as 100% survival. The concentration resulting in 50% of cell-growth inhibition (IC_50_) was calculated using SigmaPlot version 8.0 software (SPSS Inc., Chicago, IL, USA).

### Flow cytometric analysis of PI-stained cells

Propidium iodide (PI) staining and flow cytometry analysis were performed as described previously [15]. The fraction of cells with hypodiploid DNA content was considered as the apoptotic cell population. Cell cycle fractions were estimated using WinMDI Version 2.9 (The Scripps Research Institute, La Jolla, CA, USA) and Cylchred (Cardiff University, Cardiff, UK) version 1.0.2 software.

### Proteasome activity assay

The chymotryptic activity of the proteasome was estimated as described previously [15], using succinyl-Leu-Leu-Val-Tyr-AMC substrate (Bachem, King of Prussia, PA, USA). Fluorescence of the released 7-amido-4-methylcoumarin dye was measured on a SpectraFluor multiwell plate reader (Tecan, Salzburg, Austria), set at an excitation wavelength 380 nm and emission wavelength 460 nm.

### Western blot analysis

Western blot analysis was performed as described before [15]. Rabbit polyclonal antibodies used were: anti-Hsp70 (Affinity Bioreagents, Golden, CO, USA), anti-Hsp90 (Cell Signaling, Danvers, MA, USA), anti-20S proteasome core, anti-subunit β_1_, β_2_, β_5 _(Biomol, Plymouth Meeting, PA, USA), anti-Mcl-1 (clone 22, BD PharMingen, San Diego, CA, USA), mouse monoclonal antibodies used were: anti-Hsp27 (Cell Signaling), anti-β-actin (Sigma-Aldrich, St Louis, MO, USA).

## Competing interests

The author(s) declare that they have no competing interests.

## Authors' contributions

JV conceived of the studies, carried out experimental assays, analyzed data and drafted the manuscript. AC carried out experimental assays and drafted the manuscript. GG initiated the studies and drafted the manuscript. All authors read and approved the final manuscript.
